# Improved in vivo imaging method for individual islets across the mouse pancreas reveals a heterogeneous insulin secretion response to glucose

**DOI:** 10.1038/s41598-020-79727-8

**Published:** 2021-01-12

**Authors:** Henriette Frikke-Schmidt, Peter Arvan, Randy J. Seeley, Corentin Cras-Méneur

**Affiliations:** 1 Department of Surgery, University of Michigan, Ann Arbor, MI USA; 2 Division of Metabolism, Endocrinology and Diabetes, Department of Internal Medicine, University of Michigan, 2800 Plymouth Rd, Ann Arbor, MI 48109 USA

**Keywords:** Fluorescence imaging, Metabolism, Homeostasis

## Abstract

While numerous techniques can be used to measure and analyze insulin secretion in isolated islets in culture, assessments of insulin secretion in vivo are typically indirect and only semiquantitative. The CpepSfGFP reporter mouse line allows the in vivo imaging of insulin secretion from individual islets after a glucose stimulation, in live, anesthetized mice. Imaging the whole pancreas at high resolution in live mice to track the response of each individual islet over time includes numerous technical challenges and previous reports were only limited in scope and non-quantitative. Elaborating on this previous model—through the development of an improved methodology addressing anesthesia, temperature control and motion blur—we were able to track and quantify longitudinally insulin content throughout a glucose challenge in up to two hundred individual islets simultaneously. Through this approach we demonstrate quantitatively for the first time that while isolated islets respond homogeneously to glucose in culture, their profiles differ significantly in vivo. Independent of size or location, some islets respond sharply to a glucose stimulation while others barely secrete at all. This platform therefore provides a powerful approach to study the impact of disease, diet, surgery or pharmacological treatments on insulin secretion in the intact pancreas in vivo.

## Introduction

With increased insulin resistance, pancreatic β-cells adapt through hypertrophy, hyperplasia and increased insulin secretion. Failure to meet this increased demand either result from MODY or SIDD^1^, or leads to Type 2 diabetes (reviewed in^2^). Pancreatic exhaustion with depletion of insulin content have been increasingly seen as a leading trigger of islet failure^3–5^. While the overall insufficiency of insulin secretion can be measured, little is known about the response of individual islets during normal as well as pathological conditions associated with poor glucose control. Most islet studies are performed by islet purification from the remaining pancreas in order to examine responses ex vivo in culture^6^. While much can be learned from this approach^7^, we know that a wide range of extra-islet signals can have profound effects on insulin secretion. These may include neural signals^8^, nutrients^2^, hormones^9,10^, and blood flow^11^. Thus, isolated islets in culture cannot capture the full complexity that determines insulin secretion in vivo. Pancreatic slice cultures^12–14^ and islets implanted in the anterior chamber of the eye^15–19^ have more recently been developed to try and provide a more physiological and “in context” environment. Although these methods have proven immensely valuable, they are not without caveats and still differ significantly from the normal physiological environment^20^. Consequently, it is critical to understand the function of individual islets in vivo if we are to assess both the normal ability for glucose to provoke insulin secretion and the islet pathophysiology that is linked to the development of type 2 diabetes.

Several approaches to imaging individual islets in vivo have been attempted. Using laparoscopic incisions, investigators have obtained live high-resolution longitudinal imaging of individual islets^11,21,22^. This procedure can be further enhanced through the use of an imaging window to include clusters of islets^23,24^ or using a micro-stage with a stick-type water immersion lens to compensate for the inherent movement due to the breathing of the anesthetized mouse^25^. While each of these in vivo approaches has immediate applications for live islet imaging, they are limited by a restricted field of view and the limitations of cellular reporters that can be used to analyze islet function longitudinally, and by the number of islets that can be imaged in each experiment.

**Figure 1:**
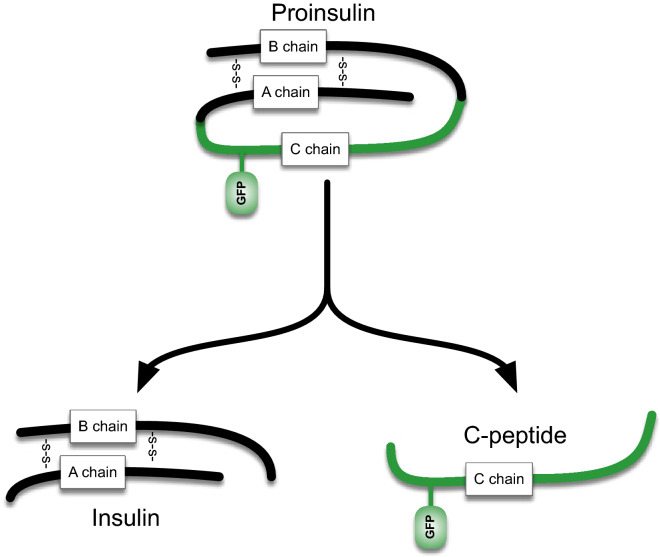
CpepSfGFP diagram. Diagram of the structure of the proinsulin with the super folder GFP (sfGFP) tag attached to it. Processing the proinsulin separates the normal insulin (left) from the CPeptide with the sfGFP tag attached to it (right).

Zhu et al*.* recently developed a mouse model with a fluorescent human proinsulin bearing a superfolder GFP-tagged C-PEPTIDE (CpepSfGFP, see Fig. 1) expressed exclusively in β-cells^26^. This model allows longitudinal insulin secretion monitoring in live anesthetized mice. The original report provided a qualitative appreciation of individual islet response based on a limited number of islets at the time, suggesting at differences in glucose response dynamics that could take place between the in vitro and in vivo conditions^26^. The original approach was relatively limited due to biological challenges like anesthesia and temperature control of the exteriorized organ—as well as technical challenges like motion blur from the breathing animal and the possibility to quantify simultaneously insulin concentrations in hundreds of individual islets over the course of a glucose challenge. We have therefore sought to adapt different methodologies to best limit the impact of each aspect, allowing a quantitative comparison of the response of individual islets in vitro and in vivo over the course of a glucose challenge. The reporter has been constructed as a transgene that produces only 0.04% of the total insulin made in the islets and does not metabolically alter the animals nor impair the normal functions of β-cells. Nevertheless, the exceptional brightness of the reporter allows for visualization of the accumulated insulin secretory granule fluorescence across the surface of the whole pancreas, within the islets where it is stored prior to secretion. In addition, CpepSfGFP is secreted alongside the normal insulin^27^, following the same dynamics. With this approach both expression and secretion of insulin in a number of individual islets can be visualized and quantified in anesthetized mice under various conditions^26^.

Described here is a large-scale effort to optimize this approach to make it more reliable, sensitive and quantifiable, tracking the response of large numbers of individual islets over time. We developed an improved methodology comparing anesthesia conditions, optimizing temperature control of the exteriorized organ and using imaging in high dynamic range and in rapid succession with tiling, combined with computer-assisted masked morphometry to overcome motion blur. This approach allowed us to monitor and quantify insulin content through the CpepSfGFP reporter simultaneously for the first time in up to two hundred individual islets in the pancreas of anesthetized mice.

We compared the visualization of insulin secretion ex vivo to a wide range of conditions to explore the response of individual islets in situ within the intact pancreas in vivo. We found that individual islet response to glucose is quite heterogeneous, emphasizing the impact of the inherent differences between in vitro and in vivo conditions and implying that a number of external factors impact individual islets response upon glucose response in vivo.

## Results

### CpepSfGFP islets respond synchronously to a glucose challenge in vitro

The CpepSfGFP reporter (Fig. 1) is a bright reporter that reflects insulin content. When stimulated with glucose, the islets secrete insulin /CpepSfGFP and the integrated density of the reporter fluorescence goes down. In their original report of the CpepSfGFP model, Zhu et al*.* were unable to detect intensity changes in isolated islets free floating in culture and with 8-bit imaging upon glucose stimulation^26^.

In order to be able to track individual islets in culture over the duration of a glucose challenge, islets were immobilized in culture in a collagen gel similarly to what has been previously used for embryonic pancreatic rudiments in culture^28–32^. The collagen is considered to be mostly inert and doesn’t affect insulin secretion. Its wide pores allow for nutrient and glucose rapid diffusion to the embedded tissue.

**Figure 2:**
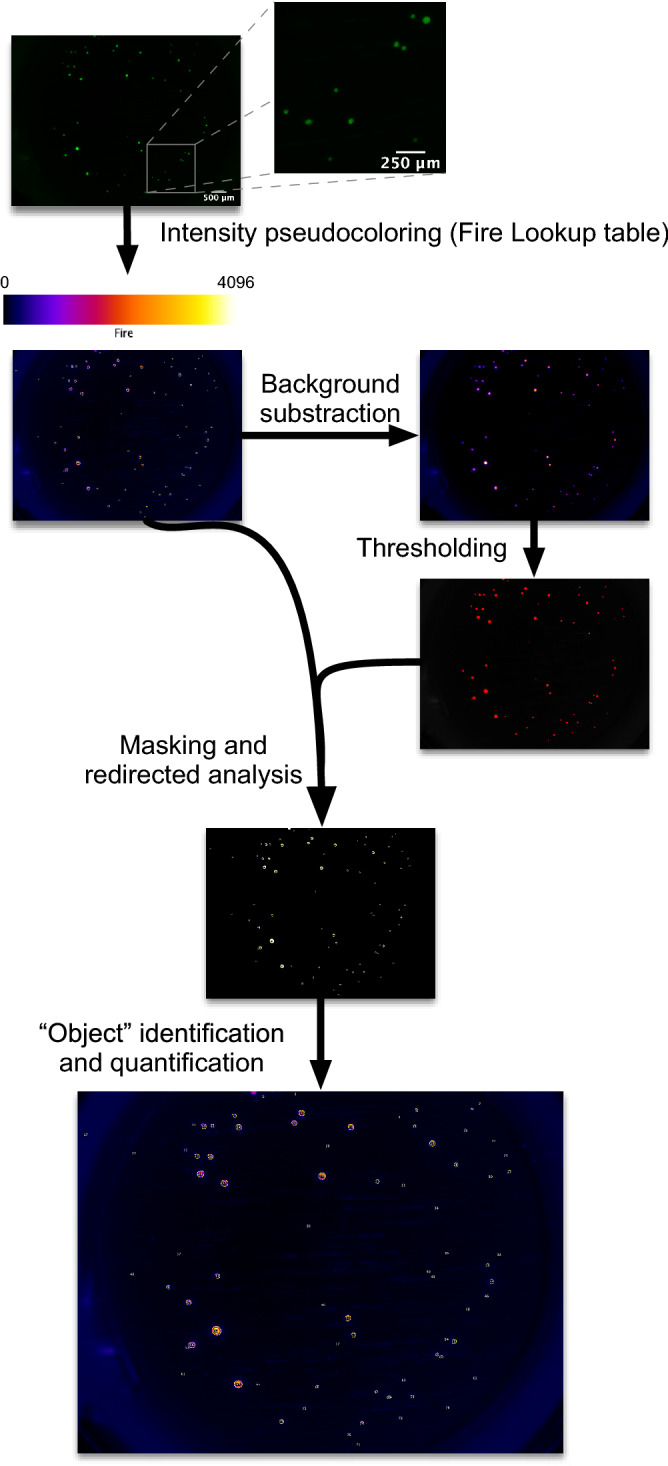
CpepSfGFP imaging on islets in culture. Flowchart of the imaging and analysis of the CpepSfGFP islets in culture. Captured images (close-up on the right) undergo pseudo-coloring using the “Fire” lookup table (scale on top of the image). Background subtraction and thresholding are used to generate a mask to be used for islet identification on the original image. Each islet or small cluster of islets (defined as “objects”) can be quantified and compared over the different time points. Scale bar on the original image: 500 μm. Scale bar on the close-up: 250 μm.

**Figure 3:**
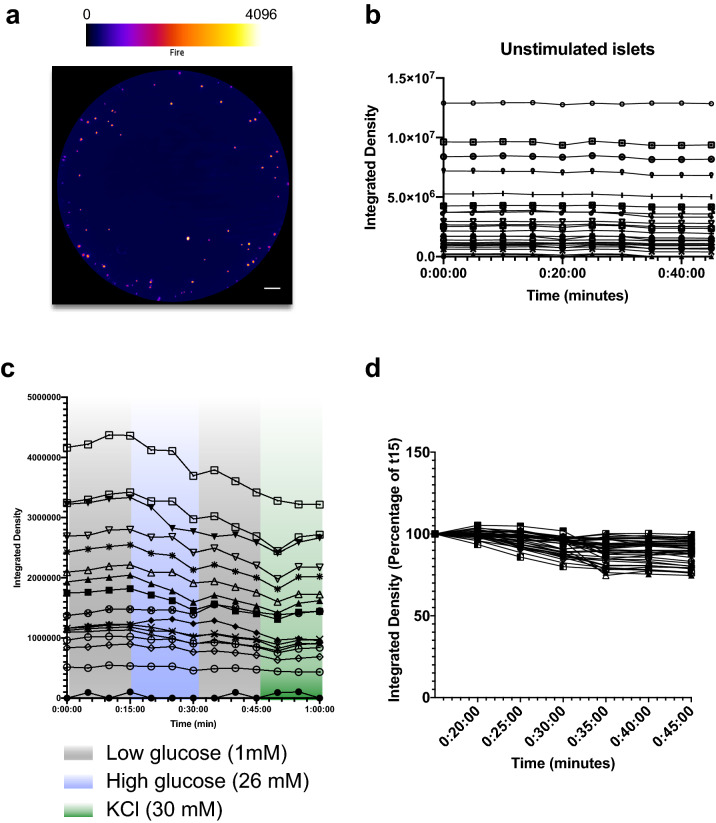
CpepSfGFP profiles in islets in culture. (**a**) CpepSfGFP islets in culture in collagen with intensity-based “Fire” pseudocoloring (scale on top). (**b**) Integrated density of the unstimulated individual CpepSfGFP islets over 45 min of culture. (**c**) Integrated density of CpepSfGFP individual islets in culture during a GSIS in low (1 mM) glucose, then high (26 mM) glucose, low glucose again and finally KCl (30 mM). (**c**) Individual islets from figure (**c**) represented as a percentage change from t15 (time at which the islets are stimulated with glucose). Scale bar 500 μm.

Islets were embedded in collagen with media added on top of the gel layer after polymerization. As illustrated on Fig. 2, fluorescence in the wells from individual CpepSfGFP islets was easily detected in islets as small as 20 μm in diameter (see close-up in Fig. 2). Using a high dynamic range camera (12-bit imaging), both low and high intensity islets could be imaged and quantified without clipping (saturation) of the signal (represented by pseudo-color with an intensity-based lookup table in Fig. 2). Islets could then be imaged every 5 min over the course of GSIS. Masking after background subtraction helped to delineate individual islets or small clusters of islets. Using these masks, the intensity of the signal on the original images derived from insulin/CpepSfGFP content in the individual islets over time were quantified throughout the experiment (represented in Fig. 2). In the experiments presented in Figs. 2 and 3, the density of islets in the well was chosen to minimize the risk of having islets aggregated together, and with our mask, all images allowed clear delineation of individual islets.

Zhu et al*.* had previously illustrated that the SfGFP reporter did not seem to change in intensity until islets were stimulated by a glucose challenge^26^. In order to further confirm stability of the signal from the reporter, CpepSfGFP islets were kept in culture in low glucose (1 mM) and imaged over 45 min. As illustrated in Fig. 3b, islets did not display any change of intensity over this period of time, regardless of their size or location illustrating that the imaging conditions did not lead to any appreciable photobleaching or other fluorescence quenching. Additionally, when challenged with high glucose (26 mM) and thereafter with KCl (30 mM), essentially all islets responded to these stimuli as measure by a loss of SfGFP signal intensity from the islets (Fig. 3c). After a 15 min challenge with high glucose, average islet SfGFP signal intensity declined ~ 10% from initial values, and after a subsequent 15 min stimulation by depolarization with KCl, the average SfGFP signal intensity declined to ~ 12.5% from starting values. A small number of islets exhibited a slight increase after KCl stimulation. As this was not a clear and reproducible pattern but rather something observed in spot time points, we restricted our analysis to the overall profiles that extended across the entire stimulation period so as to avoid over-reliance on single time points. Importantly, the response of the islets appeared independent of islet size, consistent with the notion that all islets in culture secrete a small fraction of their available insulin into the media in response to stimulation with high concentrations of glucose or potassium-mediated depolarization (Fig. 3d). The experiment was repeated on 8 independent preparations which exhibited similar patterns all within the same magnitude of response as illustrated and analyzed comparatively later in Fig. 7e,f.

### Surgical distress blunts insulin response in vivo

**Figure 4:**
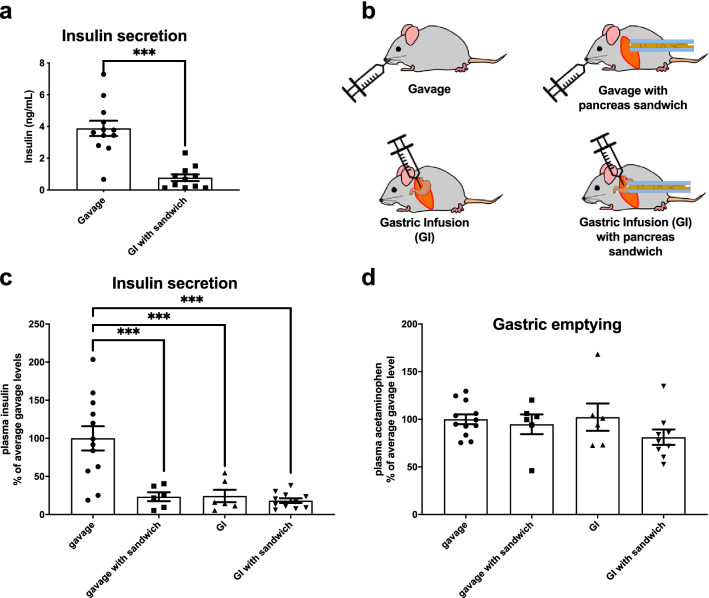
Impact of surgery. (**a**) Glucose-stimulated insulin secretion on mice stimulated by gavage or by gastric infusion after having the pancreas exteriorized. (**b)** Diagram of the different conditions used for analysis in panels (**c**) and (**d**). Insulin secretions (**c**) and gastric emptying (**d**) in mice that were glucose-stimulated by either gavage, gavage with pancreas exteriorization (sandwiching), gastric infusion and gastric infusion with pancreas exteriorization (sandwiching). ***p < 0.001.

Visualizing the CpepSfGFP islets in live mice requires a laparotomy and exteriorization of the pancreas. Any form of surgery, even under anesthesia, triggers a stress response with an increased secretion of pituitary hormones and activation of the sympathetic nervous system^33^. In a preliminary set of experiments, the insulin secretion 15 min after a glucose challenge of animals that underwent laparotomy for pancreas imaging were compared to control mice that had not undergone anesthesia or surgery. Figure 4a demonstrates that while animals that were anesthetized and had surgery for pancreas imaging still exhibited glucose-stimulated insulin secretion, the magnitude of the response was fractionally smaller than that of the reference group (non-anesthetized mice subjected to glucose stimulation through gavage).

To avoid gavaging of anesthetized mice (which poses technical challenges), we tested the use of gastric infusion by inserting a catheter into the stomach as part of the surgical procedure, prior to pancreas externalization.

In order to distinguish the effects of the surgery itself from the method of glucose stimulation, the animals were split in four groups (1) gavaged mice, (2) gavaged mice with pancreatic exteriorization, (3) mice that underwent a simple laparotomy without pancreatic exteriorization and had a glucose stimulus through gastric infusion and finally (4) mice that underwent laparotomy, pancreatic exteriorization and glucose stimulation by gastric infusion (see diagram in Fig. 4b). In all cases, regardless of the method used for glucose stimulation, all three groups that had a laparotomy had a blunted insulin secretion in response to glucose (Fig. 4c). In addition, all four groups had near identical gastric emptying rates (Fig. 4d).

### Temperature control, anesthesia and analgesia improve the magnitude of insulin secretion in response to glucose

**Figure 5:**
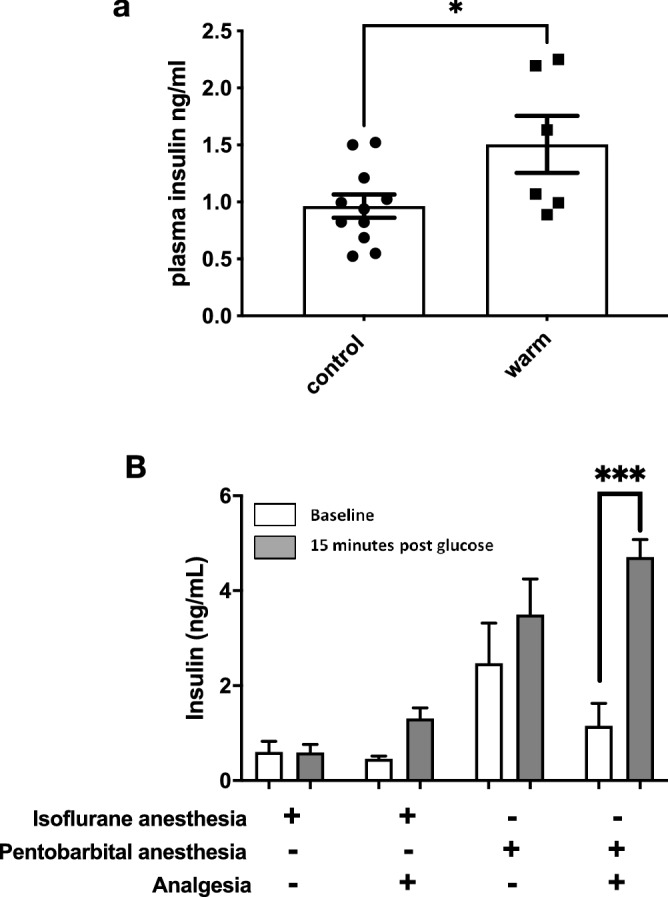
Impact of temperature, anesthesia and analgesia. (**a**) GSIS in anesthetized mice with the pancreas exteriorized and sandwiched on a slide on plastic spacers (control), or on copper tubing (warm). (**b**) Comparison of GSIS on animals with either isoflurane or pentobarbital and with or without analgesia. *p < 0.05, ***p < 0.001.

While the mice were always kept on a 37 °C heating pad during the surgical-imaging procedure, exteriorization makes the pancreas itself subject to additional heat loss. Despite pre-warming the slides to 37 °C for imaging, we noted that the temperature on the slide dropped to 22 °C within a few minutes. In order to test the possibility that this drop in temperature could blunt insulin secretion, we replaced the plastic spacers defining the pancreatic sandwich with copper tubing. Copper has a thermal conductivity of 399 W m^−1^ K^−1^ (over 400 times better than most plastics). The tubing was placed alongside the back and the abdomen of the mouse, reinforcing core temperature control on the mouse itself, and increasing the temperature on the slide to 36.8 °C (illustrated in Supplemental Fig. 1). We therefore compared insulin secretion with the plastic spacers or with copper tubing and noted a ~ 1.5-fold increase in insulin secretion (Fig. 5a).

Isoflurane is one of the most widely used anesthetics in mice. Induction and recovery are fast, and the risk of accidental death is low. Although the effects of anesthesia on insulin secretion have been previously studied^33–37^, most anesthetic agents can also blunt insulin secretion to glucose to some degree^34,35,37^. Nevertheless, pentobarbital has been reported to have less impact on glucose tolerance tests and insulin secretion^37^. We therefore compared anesthetic agents and analgesia (using copper tubing for temperature control) to further examine the impact of the laparotomy on glucose-stimulated insulin secretion in vivo. Figure 5b demonstrates that pentobarbital can in part restore proper insulin secretion compared to isoflurane, and that analgesia further ameliorates insulin secretions in response to a glucose challenge. Together, temperature control, analgesia and the use of pentobarbital for anesthesia allowed recovery of stimulated insulin secretion from the exteriorized pancreas that best resembles what is typically observed from non-anesthetized mice.

### Optimized platform for CpepSfGFP GSIS imaging in anesthetized mice

Imaging a live pancreas under fluorescence in an anesthetized mouse presents additional challenges compared to imaging islet CpepSfGFP secretion in vitro. The live animals are breathing so that the abdomen is constantly moving under the camera. This can lead to significant motion blur, complicated by the fact that the large pancreas area cannot be imaged in only one field without a dramatic loss of magnification/resolution. Indeed, in order to be able to simultaneously track as many islets as possible, the images need to be acquired with a wide depth of field, keeping as many islets as possible in focus.

**Figure 6:**
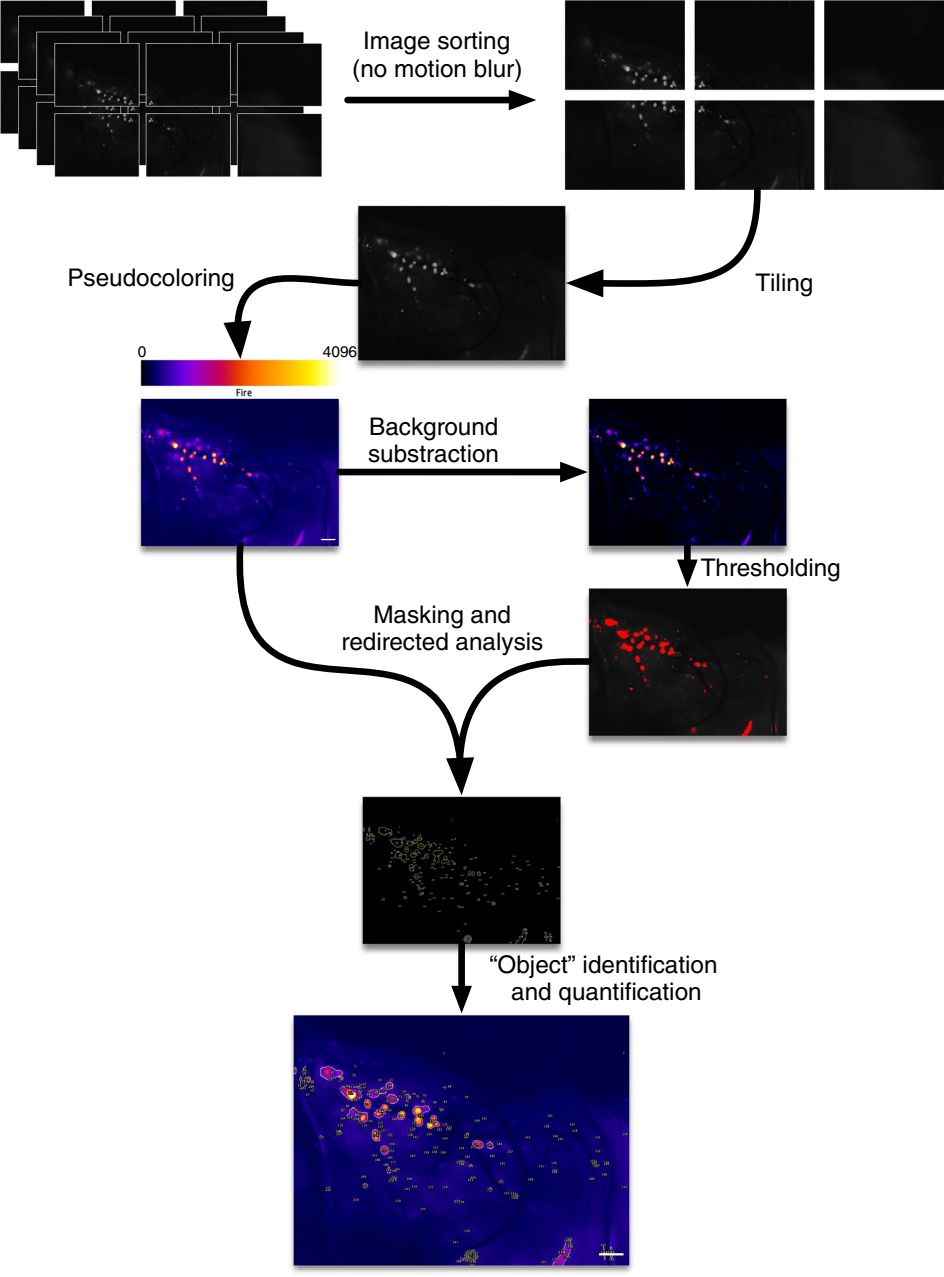
Live CpepSfGFP islet imaging. Flowchart of the imaging and analysis of the CpepSfGFP islets in vivo in anesthetized mice. Rapid-fire imaging allows the capture of multiple images in each position. The images are then filtered to eliminate the ones with visible motion blur and tiled to recompose a complete high-resolution view in 12 bit of the entire pancreas. The images undergo pseudo-coloring using the “Fire” lookup table (reference scale on top of the image). Background subtraction and thresholding are used to generate a mask to be used for islet identification on the original image. Each islet or small cluster of islets (defined as “objects”) can be quantified and compared over the different time points. Scale bar 1 mm.

To cover the entire area of the tissue, we chose to use a microscope with a motorized stage and a long working distance and objectives with adjustable aperture offering a wide depth of field and therefore allowing image capture of the entire pancreas that could be exteriorized on a motorized stage by tiling ~ 6 fields, while maintaining most imaged islets within the focus range. To minimize motion blur, we programmed the microscope to take 4 images in rapid succession from each field chosen by the motorized stage. Only one of the four images was subsequently chosen to maintain focus and synchronize the image capture with the breathing cycle of the animal. This allowed to obtain images with a resolution of 0.54 μm per pixel and reliably detect and quantify islets as small as 10 μm in diameter. Tiling allowed us to recompose a complete overview of the tissue (schematized in Fig. 6). The remainder of the imaging analysis used the same approach as that for isolated islets in vitro in collagen. Using this platform, we were able to track over the glucose challenge up to 200 individual islets, and small clusters of islets.

### CpepSfGFP islets respond heterogeneously to a glucose challenge in live, anesthetized mice

**Figure 7:**
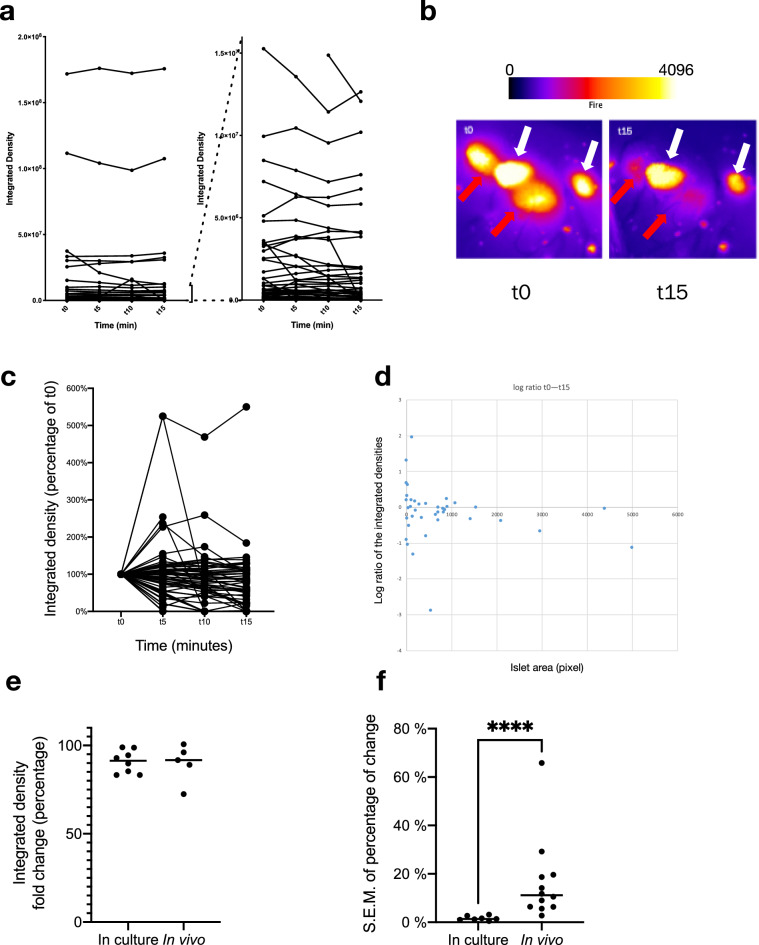
CpepSfGFP profiles on islets in vivo in anesthetized mice. Expression profiles of the CpepSfGFP islets in vivo in anesthetized mice after a glucose stimulation (gastric infusion) at t0. (**a**) Expression profile of all individual islets (left) or a subset of smaller islets (different scales on the right) over 15 min. (**b**) Pseudo-colored islets (fire lookup table, scale above) before stimulation (t0) and after 15 min (t15). The white arrows indicate islets that exhibit only modest changes in 15 min while the red arrows highlight adjacent islets that change dramatically over the same time course. (**c**) Integrated density of the individual islets expressed as a percentage at t15 relative to their value at t0. (**d**) Log ratio representation of the islet response based on the integrated density on the vertical axis and their area (expressed in pixels) on the horizontal axis. (**e**) Comparison of the total islet response to the glucose challenge in culture and in vivo (percentage change of the sum of all integrated densities in a culture well or a pancreas). (**f**) SEM of the islet response (comparing the distribution of individual islets response expressed as a percentage at t0 compared to t15) of islets in culture compared to islets imaged in vivo. ****p < 0.0001.

While isolated islets respond in a coordinated fashion in vitro, we were surprised to find that islet response to intragastric glucose is vastly heterogeneous in vivo: some islets dropped dramatically in intensity while others did not seem to respond at all (Fig. 7a). Looking at small groups of islets at a higher magnification suggested no consistent topographical impact in determining islet response to high glucose stimulation. In small groups of islets, we often saw some islets responding sharply while immediately adjacent islets exhibited little change in intensity (Fig. 7b). A representation of the islet dynamics in terms of the proportion (percentage of the changes based on t0) illustrates the wide range of response observed in vivo (Fig. 3c,d). Plotting the islet response on a logarithmic scale based on integrated density change ratio (on the vertical axis, in log scale) and area (on the horizontal axis) demonstrated no discernable relationship between islet size and insulin secretion, ruling out a size effect (Fig. 7d). A few of the small islets seemed to show actual increase in insulin content, which could relate to actual insulin biosynthesis or the fact that smaller islets are more susceptible to measurement variability inherent to the movement of the pancreas in three dimensions. As illustrated in Fig. 3d, stimulated islets in culture respond quite homogeneously with a very tight SEM (Fig. 7f summarizes the SEM in 8 independent experiments). In vivo, the islet responses are vastly more heterogeneous, with a much wider SEM (Fig. 7f, SEM in 5 different mice). Comparing the loss (of integrated density) of islet CpepSfGFP upon high glucose stimulation in vivo, the overall response of all islets together averaged ~ 8% of total insulin/CpepSfGFP content, which was quite similar to that seen in vitro (Fig. 7e, 7 in vitro experiments vs 12 independent mice). However, the comparison of the SEM of the responses of individual islets in culture and in vivo clearly reflected a greater heterogeneity under the latter condition (Fig. 7f).

## Discussion

The development of type 2 diabetes results from reductions either in islet mass, function, or both. Dramatic changes in islet mass can affect the long-term risk of developing diabetes. Pancreatic morphometry after immunostaining has been a long-established gold standard to assess β-cell mass. While the dynamics of the coordination of individual β-cells within individual islets has been well documented^38^, very few functional tests have been available for in vivo studies, in particular when attempting to address individual islet responses throughout the whole pancreas. In 1987, Stefan et al*.* sampled the pancreas by immunostaining after a glucose challenge and hypothesized that insulin secretion was heterogeneous within an islet, as well as between islets, depending upon where they were located^39^. Their approach was purely qualitative and provided limited sampling, but it was a first attempt to address potential heterogeneity of insulin secretory responses of islets in vivo.

In 2016, Zhu et al*.* described a mouse model that could allow monitoring of insulin storage and secretion in vivo and in vitro^26^. The original method allowed for the rudimentary analysis of the dynamic of secretion for groups of islets at the time as well as important qualitative aspects. However, we discovered, and devised means to circumvent, multiple technical obstacles that affect insulin secretory responsiveness in vivo, and the ability to accurately image and quantify a wide range of the insulin content within multiple islets across the surface of the pancreas over a time course. Here, we have presented a means to significantly improve the original platform to longitudinally monitor hundreds of islets at a time in vivo in anesthetized mice. Using this platform, we have been able to visualize for the first time the differences in the response of islets in vivo compared to what had been described using in vitro approaches. While islets in vitro more consistently secrete a small fraction of their individual insulin content in response to high glucose, islets in vivo display drastically different responses throughout the pancreas (Fig. 7).

Investigators have previously imaged secretion from live individual islets^22^, or groups of islets^22^ in a number of limited and indirect ways. Other models have helped to assess islet physiology in various physiological contexts. For instance, Ca^2+^ flux in response to a glucose challenge has also been studied in pancreatic slices^13^ or in vascularized islets implanted in the anterior chamber of the eye^40^. In this manuscript, we have used a fluorescence reporter that allowed us to directly monitor insulin/CpepSfGFP secretion in up to two hundred individual islets at a time, repeatedly, throughout a glucose challenge in a live animal where the islets still maintain their native vascularization and innervation.

We note several caveats to our studies. Exteriorizing the pancreas allows in vivo imaging for most of the body and tail of the pancreas but imaging the head of the pancreas is not physically possible without significant mechanical stress on the duodenum and stomach. In addition, smaller islets that are embedded deep within the pancreas are less likely to be imaged. In order to minimize this problem, we used an objective with a wide depth of field, maximizing the number of islets that are within focal range. The SfGFP reporter is extremely bright and allows capture of islets that could be deeper from the pancreatic surface, with the caveat that the thickness of overlying non-islet tissue above can weaken fluorescence signal, diffuse the emitted light, and thus blur the shape of the islets. In addition, the mice are anesthetized and breathing, leading to movements of the pancreas. Having the pancreas resting on a heated slide somewhat limits the magnitude of movements of the tissue, but it could still affect imaging of individual islets. While large islets are mostly unaffected by these small movements in 3D, the effect is not always negligible on smaller islets. These caveats do not affect the overall integrated density of the captured images, although they can affect the precise estimation of islet size and the degree of intensity changes on the smallest islets, which could explain why some of the smallest islets appear to increase in intensity over the course of a glucose challenge.

Using a heated slide (raised on heated copper tubing) with spacers between the slide and coverslip helped to restrict slide motion and improved secretory responsiveness. Rapid-fire imaging (succession of captures in a reduced time frame) maximized the chance to capture images free of motion blur throughout the animal breathing cycle, with a motorized stage used for tiling higher-resolution images of the pancreas. Moreover, we used high dynamic range imaging (12-bit, 4096 levels of intensity) to capture without saturation the faintest as well as the brightest islets. In addition to maximizing the range of intensities of islets that could be captured, the increase of dynamic fluorescence range increased the number of usable imaged islets that could be captured without saturation of the signal.

Islet response in vitro has been extensively studied and islets are known to respond in a coordinated manner, secreting only a small fraction of their insulin content^41^. The platform described in this report allows for the first time the ability to follow differences in the responses of individual islets in vivo in anesthetized mice. Notably, while some islets respond acutely, others seem unaffected by the glucose challenge. Islet distribution is similar in mice and human^42–46^, scattered throughout the head, tail and body of the pancreas. Using crude immunological assessments, it had been hypothesized that some islets might respond better than others based on their location within the pancreas itself^39^. However, our data does not support this hypothesis since islets in very close proximity to one another were found to display dramatically different responses (see Fig. 7B). Moreover, the close proximity of islets exhibiting drastically different secretory responses would also seem to discount the possibility that mechanical stresses blunt the response across an entire region of the pancreas. Alternatively, it has been hypothesized that differences in response between islets could result from innervation, vascularization or that some islets are inherently stronger in their secretory response to glucose^39^. It has been demonstrated that insulin can inhibit its own secretion^47–50^. Conceivably, the first islets to sense a rise in blood glucose could secrete larger amounts of insulin, thereby inhibiting responses of islets further downstream. However, this hypothesis remains untested. Further, if innervation controls the magnitude of the response to glucose, denervating the pancreas should theoretically restore homogeneity to responses between islets.

**Figure 8:**
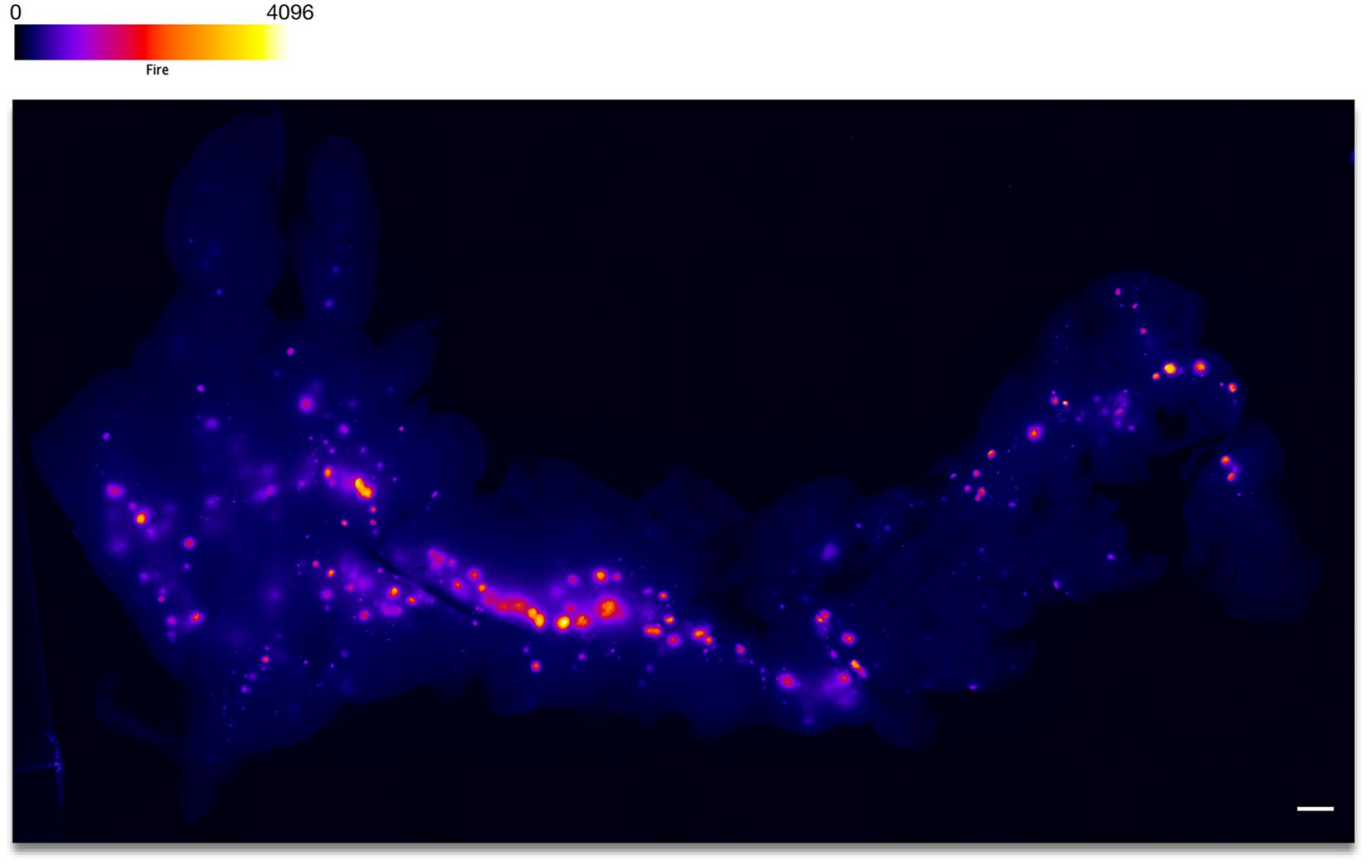
Excised pancreas from a CpepSfGFP mouse. Fluorescence imaging with intensity-based “Fire” pseudocoloring (scale on top) of an excised pancreas from a CPepSfGFP mouse. The tail of the pancreas is on the left and the head on the right. Scale bar 1 mm.

A number of limitations and technical challenges remain. Our platform is mostly suited for small mammals. Only the tail of the pancreas can be safely imaged without inducing tissue damage to the internal organs. For comparison, Fig. 8 shows an excised pancreas from a CPepSfGFP mouse, illustrating the distribution of islets between the tail (on the left) and head (on the right) of the pancreas. And while the quantification of the integrated density remains tolerant to some changes in focus, small islets far from the surface are likely not being captured. This implies that even though we can capture and quantify hundreds of islets at the time, we are only quantifying a subset of all the islets in the pancreas. Additionally, while our approach is well adapted for the study of the heterogeneity of individual islets responses, it cannot achieve a spatial resolution that could allow for the analysis of heterogeneity within individual β-cells. A number of limitations and technical challenges remain. Only the tail of the pancreas can be imaged by our methodology without inducing tissue damage to other internal organs. For comparison, Fig. 8 shows an excised pancreas from a CPepSfGFP mouse, illustrating the distribution of islets between the tail (on the left) and head (on the right) of the pancreas. And while quantification of the integrated density remains tolerant to some changes in focus, small islets far from the surface are almost certainly not being fully captured. This implies that even though we can capture and quantify hundreds of islets at a time, we are only quantifying a subset of all the islets that reside in the pancreas in situ. Additionally, while our survey approach is well adapted for the study of the heterogeneity of individual islets responses, it cannot simultaneously achieve a spatial resolution that allows for the analysis of heterogeneity within single β-cells within individual islets.

Described here, however, is a platform that allows for the testing of various hypotheses about what contributes to the heterogeneity of islet insulin secretion responses in vivo. Moreover, islets can be compared in the normal state, or under pathological conditions, or under the influence of various pharmacological agents, nutrients, infused antibodies, or surgical intervention. The CpepSfGFP mice can also be crossed with other loss or gain of function genetic models in order to test specific molecular hypotheses. The platform described here constitutes a robust and practical integrative physiology model that can be used to compare insulin secretion at the level of the islet in the physiological and pathophysiological in vivo milieu, and while we chose to image track and quantify islet response during the first 15 min after a glucose challenge (when most of the insulin is known to be secreted), the animals can be imaged for significantly longer if need be. It can also be used in vitro under controlled conditions, and at even higher resolutions to study the secretory patterns of individual β-cells within islets.

## Methods

### Animals

All animal experiments were approved by the Institutional Animal Care & Use Committee at University of Michigan (Animal Use Protocol # PRO00007908). All experiments were performed in accordance with relevant guidelines and regulations.

The sfGFP mice were generated as described previously^26^ and maintained on a C57BL6/J background. All experiments were performed on male adult mice of approximately 3 months of age. The mice were group-housed and fed standard chow (5LOD irradiated diet, Labdiet, St. Louis, MO, USA). In every round of experiments involving a glucose tolerance test or insulin secretion tests, mice were fasted overnight with food being removed prior to lights out the evening before.

### Islet isolation

Islet isolation was accomplished by collagenase digestion based on the procedures previously described for rat islets^51^ with some modifications based on^52^. Collagenase was injected into the common bile duct clamped at the ampulla of Vater. After digestion at 37 °C, a series of low-speed centrifugation steps were performed in HBSS-10% FBS to stop the digestion. The pellet was then filtered through a strainer and centrifuged in Histopaque-1077 (Millipore Sigma, St Louis, MO, USA) under a layer of HBSS. Islets were pipetted from the Histopaque/HBSS interface and hand-picked under an inverted microscope.

### Cell culture and glucose-stimulated insulin secretion in vitro

After isolation, the islets were kept four days in culture at 37 °C with 95% humidity in media (RPMI-1640 with L-Glutamate, 10% FBS, Penicillin/Streptomycin, Minimum Essential Medium (MEM) non-essential amino acids, HEPES (Gibco, Waltham, Massachusetts, USA]). For imaging, islets from four mice were pooled and redistributed in four different wells (50 islets per well). The islets were embedded within a thin layer of 500 μL of Rat Type I collagen (Corning, Bedford, MA, USA), as previously described^29,31^.After polymerization, the gel was overlaid with 500 μL of culture media and allowed to rest for an additional day before stimulation and imaging.

### Islet glucose stimulation and imaging

Prior to glucose stimulation, the media for the islets in collagen was replaced with HEPES-buffered, Kreb-Ringer solution with 1 mM glucose for 3 h, replacing the media after 1.5 h. The plates for the islets were placed on a mat heated at 37 °C on a H101A ProScan motorized stage (Prior Scientific Instruments Ltd. Cambridge, United Kingdom) under a Nikon AZ100 multizoom microscope (Nikon Instruments Inc., Melville, NY, USA) using the AZ-Plan Fluor 5 × objective with a 4 × secondary zoom factor. The wells with the CpepSfGFP islets in collagen were then imaged before stimulation and then every 5 min throughout the experiment with a CoolSNAP (Teledyne Photometrics, Tucson, AZ, USA) 12-bit cooled CCD camera, through the NIS-Elements AR software (version 5.0.2; Nikon Instruments Inc. Melville, NY, USA). After 15 min of imaging in low glucose, the medium was then replaced with 500 μL of HEPES-buffered, Kreb-Ringer solution with 52 mM glucose to bring the final concentration of glucose (media + gel) to 26 mM. After 15 min, the medium was replaced again with 1 mM glucose HEPES-buffered, Kreb-Ringer solution for 15 min, and then, finally with HEPES-buffered, Kreb-Ringer solution with 60 mM KCl (to bring the final concentration of media + gel to 30 mM KCl). At the end of each step, media were collected, stored at − 80 °C, and insulin measured by ELISA.

### Islet imaging analysis

In order to perform the analysis of the CpepSfGFP reporter in the stimulated islets over time (in vitro or in vivo), we had to establish a multistep succession of instructions to pilot Fiji (version 2.0.0-rc-69/1.52p), a distribution of ImageJ^53–55^.

The high dynamic range 12-bit images from the experiment were analyzed directly in Fiji. As illustrated in Fig. 2, each image was duplicated. One of the images underwent background subtraction using the rolling ball method, and thresholding to create a mask. The measurement settings were adjusted to redirect the collection of the integrated density of the areas thresholded on the mask on the original unaltered copy of the image. “Particle” analysis in Fiji allowed the quantification of the integrated density (sum of the intensity of all the pixels in the area delimited by the mask) the of individual islets or small groups of islets, displaying the corresponding label on each image (see flowchart in Fig. 2). The same process was repeated for each image of individual wells throughout the experiment. All the data collected could then be parsed in a FileMaker database (version 17, Claris International Inc. Santa Clara, CA, USA). Using the labels displayed in the image, the islets were visually mapped from image to image, allowing to create a relational database of all the data (Supplemental Fig. 2) and a trace of the individual islet intensity over time. Through the relational database, the data output could then be simply tabulated and graphed, providing a trace of the integrated density of each individual islet over time, as illustrated in Fig. 3.

### Insulin measurements

Insulin was measured using the Ultra-Sensitive Mouse insulin ELISA Kit (Crystal Chem, Elk Grove Village, IL) according to the manufacturer specifications.

### Analgesia and anesthesia

Ostilox was used for analgesia at a dose of 0.1 mg kg^−1^ administered two hours prior to the individual procedure. Anesthesia was either isoflurane or pentobarbital (Euthasol Virbac AH, Inc., Fort Worth, TX) given in an induction dose of 81 mg kg^−1^ followed by a maintenance dose of 20 mg kg^−1^ given every 20 min until the end of the procedure. Acetaminophen (Sigma Aldrich, St. Louis, MO) was administered at a dose of 0.1 mg kg^−1^ for gastric emptying measurements.

### Surgical procedures: sandwiching the pancreas

After being anesthetized, the mouse was placed on a heating pad maintained at 37 °C. The left side of the mouse was shaved and placed in between 2 hollow copper tubes of. 1.5 cm of diameter. Copper conducts heat efficiently, and the tubes prevent heat loss from the sides of the mouse. They also served as a heated platform supporting the microscopy slides above the animals. An incision was made just above the spleen but below the level of the diaphragm. The pancreas was gently exteriorized and placed on the warmed microscopy slide resting on the copper tubes. It was ensured that the pancreas was flat and in a single layer across the slide. Plastic spacers 3 mm thick were placed on each side of the pancreas. Hypromellose ophthalmic demulcent solution (2.5%) (Goniovisc; HUB Pharmaceuticals, Plymouth, MI, USA) warmed at 37 °C was applied to the surface of the pancreas before a coverslip was placed over the spacers, “sandwiching” the pancreas at uniform thickness between the slide and coverslip (see diagram in Fig. 4). The gel prevents tissue drying while offering optimized light conductance for the sfGFP as previously illustrated^56^.

The heating pad with the mouse was then placed on top of the H101A ProScan motorized stage (Prior Scientific Instruments Ltd. Cambridge, United Kingdom) under the Nikon AZ100 multizoom microscope (Nikon Instruments Inc., Melville, NY, USA) for imaging (see Supplemental Fig. 1). Tail vein blood was obtained for glucose measurements and plasma insulin.

### Whole pancreas live imaging

The pancreas was subsequently also imaged on the AZ100 microscope using the AZ-Plan APO 0.5 × objective with a 4 × secondary zoom factor, and capturing using a CoolSNAP (Teledyne Photometrics, Tucson, AZ, USA) 12-bit cooled CCD camera, through the NIS-Elements AR software (version 5.0.2; Nikon Instruments Inc. Melville, NY, USA). The objective (5 ×) and secondary magnification (1 ×) were chosen to maximize the depth of field and fluorescence transmission. At this magnification, the imaging of the entire pancreas required tiling. To increase the chances of capturing without motion blur, NIS-Elements AR was programmed to capture 5 images per field in 12 bit, 250 ms apart before moving to the next field. Tiling was acquired with a 15% overlap.

After the baseline imaging, a flat dose of 75 mg glucose was administered either by gavage or intra-gastric injection through a catheter placed distally from the incision. Every 5 min the pancreas was imaged followed by blood glucose measurements. The procedure ended at 15 min after glucose administration, at which time blood was obtained for plasma insulin measurement as well. The mouse was euthanized by cervical dislocation.

### CpepSfGFP image analysis and islet quantification

The images were extracted from the NIS container using NIS Viewer 4.11.0 (Nikon Instruments Inc. Melville, NY, USA) and then reviewed in Fiji individually to select only one image without motion blur per field (see flowchart in Fig. 6). All the images of a mosaic were then reassembled in Fiji using the Grid/Collection stitching plugin from Stephan Preibisch, freely available on the ImageJ repositories (https://imagej.net/Image_Stitching). Similar to what was described above for in vitro islet imaging, the image underwent the same analysis process: duplication, background subtraction, thresholding, redirection of the particle analysis on the original unaltered image to quantify individual integrated densities and labelling before collecting and relating all data in a FileMaker database to output the integrated density of each individual islet (or cluster of islets) over time throughout the glucose challenge. Any suspicion of technical artifacts (close proximity to the edge of the slide, imaging issues, proximity of a bubble in the optical gel, etc.) led to the elimination of the islet from further analysis.

### Statistical analysis

Comparisons that passed a normality test were assessed through paired or unpaired t-tests. If normality tests failed, comparisons were analyzed using a Mann–Whitney test for unpaired data. For multiple comparisons, we used either 1-way ANOVA with Tuckey’s multiple comparison test, or 2-way ANOVA following the Holm-Šídák method.

## Supplementary Information

Supplementary Information
