# Supplementary material for: Improved in vivo imaging method for individual islets across the mouse pancreas reveals a heterogeneous insulin secretion response to glucose
Source: Sci Rep. 2021 Jan 12;11:603. doi: 10.1038/s41598-020-79727-8 (PMC7804140; doi:10.1038/s41598-020-79727-8)

Improved *in vivo* imaging method for individual islets across the mouse pancreas reveals a heterogeneous insulin secretion response to glucose

Henriette Frikke-Schmidt[1], Peter R. Arvan[2], Randy J Seeley[1,2], Corentin Cras-Méneur[2]

[1]University of Michigan, Department of Surgery, Ann Arbor, MI USA

[2]University of Michigan, Department of Internal Medicine, Division of Metabolism, Endocrinology & Diabetes, Ann Arbor, MI USA.

Supplemental figures' legends

Supplemental figure 1: Anesthetized mouse with exteriorized pancreas for imaging.

a. The anesthetized mouse is placed on a heating pad between copper tubing to preserve heat. The laparotomic incision allows for the exteriorization of the pancreas, which is placed on a slide, heated by the copper tubes, with spacers and imaging gel, and a coverslip. An elastic band helps maintain the setup from moving on the heating pad. **b**. The heated pad with the mouse can then be placed directly on the motorized stage of the AZ100 microscope.

Supplemental figure 2: Diagram of the relational database generated for the time course comparison

Schematic representation of the relational database allowing the identification of the islets at different time points and the export of the corresponding integrated densities for each islet over time.

The database integrates comparisons right before (t0) and right after (t0p) glucose infusion as well as at every subsequent time point. Each object gets an identifier in FIJI. A table of manually tracked objects gets generated to match the objects from time points to time points and allows the tables of integrated densities at each time point to be put in relation in the database.

Supplemental Fig. 1

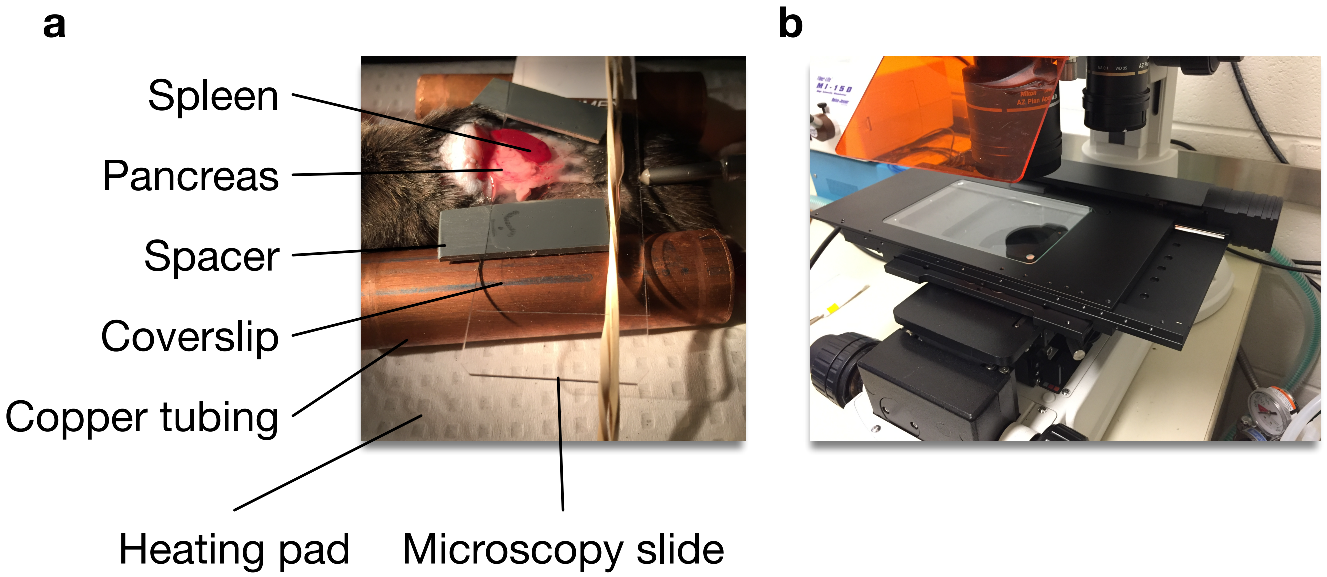

Supplemental Fig. 2

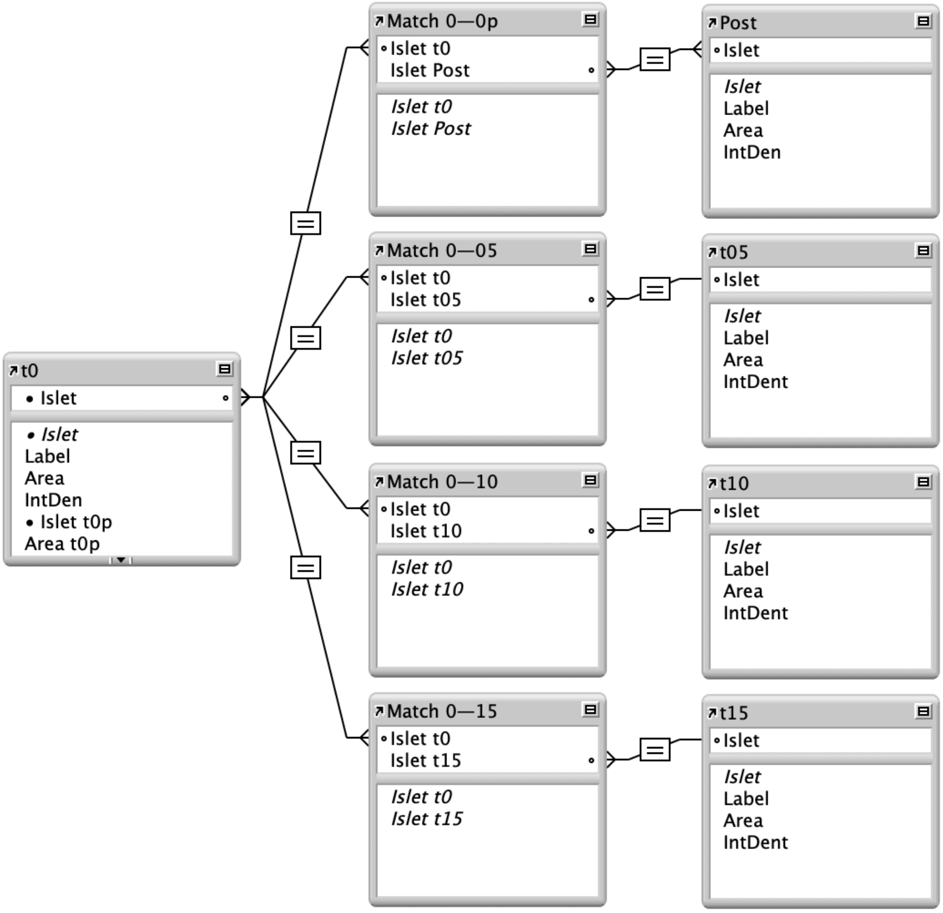

Supplement: Supplementary file 1 — Supplementary Information [file 41598_2020_79727_MOESM1_ESM.pdf]
